# Association between weight-adjusted-waist index and female infertility: a population-based study

**DOI:** 10.3389/fendo.2023.1175394

**Published:** 2023-08-08

**Authors:** Zujun Wen, Xiang Li

**Affiliations:** Department of Pharmacy, Heyuan People’s Hospital, Heyuan, China

**Keywords:** infertility, weight-adjusted-waist index, obesity, non-linear, NHANES

## Abstract

**Aims:**

Obesity is detrimental to infertility. The association between weight-adjusted-waist index (WWI, a recently developed adiposity indicator) and infertility has not previously been confirmed.

**Methods:**

The data for this study were obtained from the National Health and Nutrition Examination Survey (NHANES) 2013-2018. Each participant’s WWI was calculated as their waist circumference in centimeters by the square root of weight in kilograms. Multivariable logistic regression and generalized additive model were utilized to investigate the relationship between WWI with infertility. We used smoothed curve fitting to explore the non-linear relationship. Subgroup analysis and interaction tests were also conducted.

**Results:**

A total of 3,526 participants with ages from18 to 45 were enrolled, 364 of whom were infertile. With the higher WWI, infertility was more prevalent (OR = 1.42, 95% CI: 1.22-1.65), and this association was still consistent in subgroups (all P for interaction> 0.05). Smoothed curve fitting showed a positive non-linear relationship between WWI and infertility. Furthermore, we discovered that WWI had a stronger connection with the risk of infertility than other markers of obesity including WC, body mass index (BMI) and a body shape index (ABSI).

**Conclusions:**

Weight-adjusted-waist index levels were positively linked to an increased risk of infertility in American females and showed a stronger association than other markers of obesity. Our research indicated WWI could help identify women with infertility, and managing obesity as determined by WWI may help to reduce the risk of infertility.

## 1 Introduction

Infertility is the inability to conceive after routine unprotected sexual activity without the use of contraception for more than 12 months (1, 2). About 7% to 15.5% of women of reproductive age struggle with infertility in the United States (3, 4). It is reported that millions of families around the world, touching roughly one in seven couples in developed countries and one in every four couples in developing countries, were affected by infertility (5). Although infertility is a public health issue of worldwide concern, its determinants have not been fully elucidated.

Obesity, an increasingly widespread health burden in modern society, is increasingly common among women of reproductive age (6–8). This is pertinent since obesity is associated with certain risks, including irregular menstruation and endometrial pathology (9, 10). Obesity has been linked to an increased risk of prenatal diseases such as gestational hypertension and gestational diabetes (11, 12). Body mass index (BMI) was the traditionally recognized benchmark for determining obesity, however, it was unable to differentiate between lean mass and fat mass (13, 14). In 2018, Park et al. suggested a novel anthropometric index entitled the weight-adjusted-waist index (WWI), which can maximize the gains of WC and minimize the association with BMI (15, 16). Therefore, the weight-adjusted-waist index (WWI) reflects weight-independent centripetal obesity. Although WWI is a better predictor of obesity than BMI, its association with infertility has not been previously investigated.

In order to raise awareness of the detrimental consequences of obesity on infertility, it is imperative to identify the association between obesity assessed by WWI and infertility. Therefore, we used the National Health and Nutrition Examination Survey 2013–2018 (NHANES) to find out the association between WWI and infertility in the US female. We hypothesized that the prevalence of infertility was positively linked with the WWI level.

## 2 Materials and methods

### 2.1 Data source

The data for this study were obtained from the National Health and Nutrition Examination Survey (NHANES), a national program to assess the state of nutrition and health in the United States, which published by the National Center for Health Statistics (NCHS). To generate a nationally representative sample of non-institutionalized Americans, it was carried out using a sophisticated multistage probability design (17). An in-home interview was conducted by participants to collect data on their health, socioeconomic status, and other factors. A movable examination facility served as the setting for physical and laboratory exams.

All NHANES study methods were approved by the NCHS’s Research Ethics Review Board, and all survey respondents provided their written informed permission. On the website www.cdc.gov/nchs/nhanes/, all detailed NHANES research designs and data are accessible to the public. This cross-sectional study adhered to the STROBE (Strengthening the Reporting of Observational Studies in Epidemiology) reporting standards (18).

### 2.2 Study population

**Figure 1:**
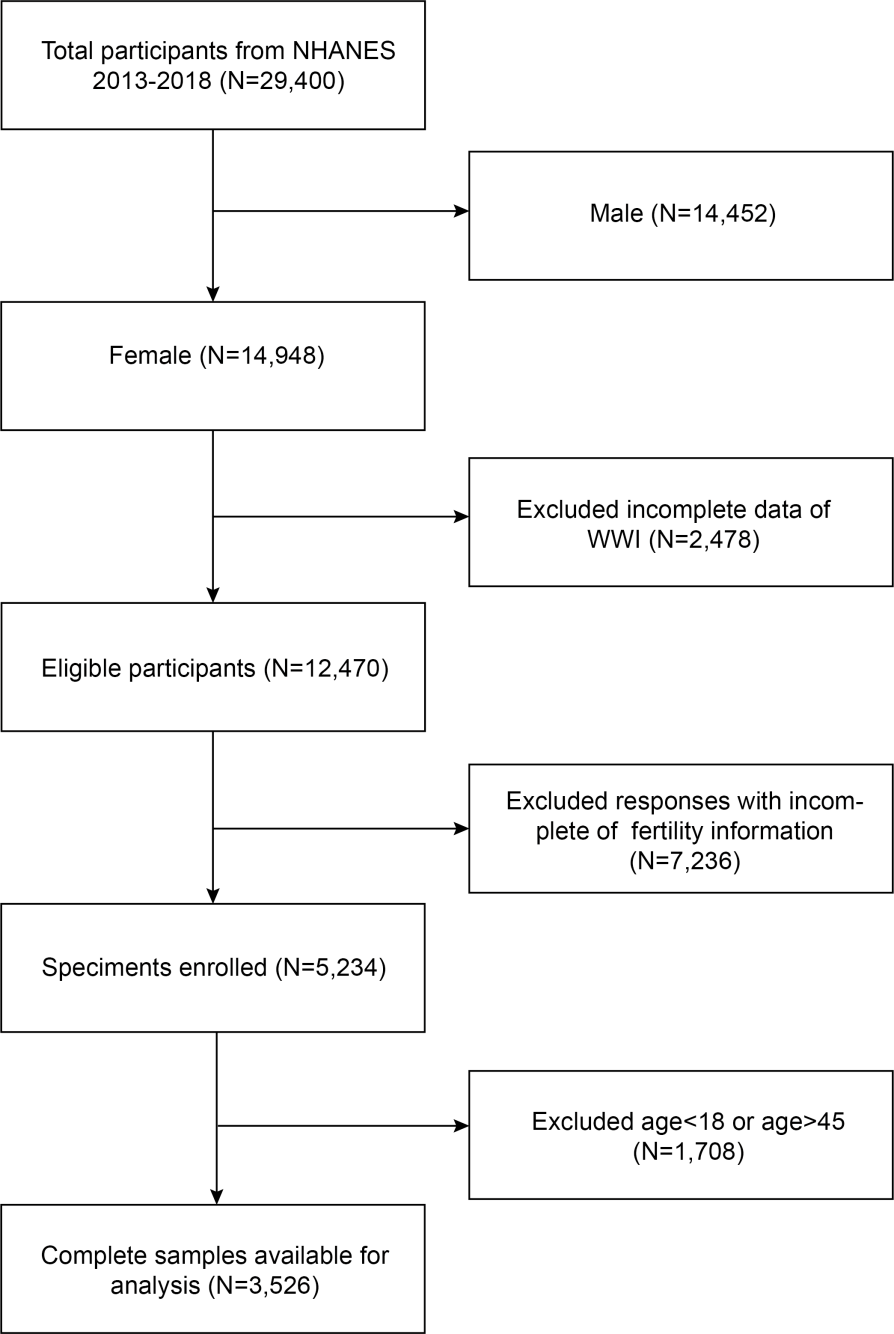
Flowchart of the sample selection from NHANES 2013–2018. A total of 29,400 participants were enrolled. After excluding male participants (n=14,452), those lacking data about the WII (n = 2,478), fertility information (n = 7,236), and women older than 45 and younger than 18 (n= 1,708), 3,526 eligible participants were included in our final analysis.

Infertility-related health questions were only included in the NHANES cycles from 2013 to 2018; hence, we used those cycles for our data. We included participants in our analysis who had comprehensive information regarding the infertility and WWI. Initially, a total of 29,400 participants were enrolled. After excluding male participants (n=14,452), those lacking data about the WII (n = 2,478), fertility information (n = 7,236), and women older than 45 and younger than 18 (n= 1,708), 3,526 eligible participants were included in our final analysis (**Figure 1**).

### 2.3 Assessment of weight-adjusted-waist index

The WWI is a WC and weight-based anthropometric measure used to evaluate obesity. An increased level of obesity was indicated by a higher WWI score. Health technicians with training in body measurements took measurements of WC and weight in a mobile examination center (MEC). Each participant’s WWI was calculated as their waist circumference in centimeters divided by the square root of weight in kilograms. Participants were grouped according to their WWI tertiles for subsequent analysis, and additionally WWI was treated as a continuous variable in the analysis. In our study, WWI was intended as an exposure. Meanwhile, we used alternative obesity indices including WC, body mass index (BMI) and a body shape index (ABSI) as the exposures. Krakauer NY & Krakauer JC defined A Body Shape Index (ABSI) as $\frac{\text{WC}}{{\text{BMI}}^{2/3}{\text{height}}^{1/2}}$ (19). To avoid having large numbers for the regression coefficients of ABSI, we multiplied the ABSI values by 1000.

### 2.4 Assessment of infertility

Each woman’s self-report from the Reproductive Health Questionnaire served as the dependent variable for infertility (variable name in questionnaire: RHQ074). The researchers probed subjects with queries such as, “Tried for a year to become pregnant?” (20). If the answer was “yes,” it signaled an “infertile” situation; if not, it indicated that it was “fertile.”

### 2.5 Assessment of covariates of interest

Our study also took into account other factors, such as age (years), ratio of family income to poverty, race (Mexican American/other Hispanic/non-Hispanic White/non-Hispanic Black/other races), education level (less than high school/high school/more than high school/others), marital status (married or living with partner/living alone), smoked at least 100 cigarettes (yes/no), had at least 12 alcohol drinks/1 year(yes/no), age when first menstrual period occurred (Age<10/10≤Age<15/15≤Age ≤ 20), had regular periods in past 12 months (yes/no), ever treated for a pelvic infection/pelvic inflammatory disease (yes/no) and ever taken birth control pills (yes/no), which may have an impact on the relationship between WWI and infertility. The complete measurement procedures for these variables were made accessible to the public at www.cdc.gov/nchs/nhanes/.

### 2.6 Statistical analysis

In accordance with Centers for Disease Control and Prevention recommendations, all statistical analyses were carried out using the proper NHANES sampling weights and took into consideration intricate multistage cluster surveys.

In descriptive analyses, the two compared groups determined by infertility status were assessed using either a weighted Student’s t-test (for continuous variables) or a weighted Chi-square test (for categorical data). Categorical parameters are presented as proportions, whereas continuous variables are summarized as means with standard deviation. Multivariable regression models that took the NHANES complex sampling design (sampling weights) into account were utilized to investigate the association between WWI and infertility. In Model 1, covariates were not adjusted at all. In Model 2, age and race were adjusted. Model 3 were adjusted for age, ratio of family income to poverty, race, education level, marital status, smoked at least 100 cigarettes, had at least 12 alcohol drinks/1 year, age when first menstrual period occurred, ever treated for a pelvic infection/PID, ever taken birth control pills, had regular periods in past 12 months. Stratified multivariable logistic regression models with stratified covariates such as age, race (Mexican American/other Hispanic/non-Hispanic White/non-Hispanic Black/other races), education level (less than high school/high school/more than high school/others), marital status (married or living with partner/living alone), ratio of family income to poverty, smoked at least 100 cigarettes (yes/no), had at least 12 alcohol drinks/1 year(yes/no), had regular periods in past 12 months (yes/no) and age when first menstrual period occurred (Age<10/10≤Age<15/15≤Age ≤ 20) were used to conduct subgroup analysis. To assess its robustness, the continuous variable WWI was transformed into a categorical variable (tertiles) for sensitivity analysis. In order to address the non-linearity of WWI with infertility and each stratification, we additionally employed a generalized additive model (GAM) and smooth curve fittings. A two-piecewise linear regression model (segmented regression model) was used to fit each period and determine the threshold effect if a non-linear connection was showed. If a non-linear association was found, the threshold effect was determined by fitting each interval with a two-piecewise linear regression model (also known as a segmented regression model). The two-step recursive technique was applied to find the breakpoint (K) that connects the segments, which was based on the model that yields the maximum likelihood.

Similarly, the smooth curve fittings were employed to identify the non-linearity of BMI on infertility, and a two-piecewise linear regression model was utilized to further evaluate their threshold effects. All analyses were performed using R version 3.4.3 (http://www.R-project.org, The R Foundation) and Empower software (www.empowerstats.com; X&Y solutions, Inc., Boston, MA). *P*<0.05 was considered as the statistically significant level.

## 3 Results

### 3.1 Baseline characteristics of study participants

A total of 3,526 participants with ages from18 to 45 were enrolled, 364 of whom were infertile. Based on their infertile status, the research participants’ characteristics are shown in **Table 1**. Self-reported infertility was more prevalent in women who were older, had higher income to poverty ratios, were married/cohabiting, had smoked at least 100 cigarettes, had been treated for pelvic infection/PID, had taken the pill, or had higher weight, waist circumference and BMI. Besides, self-reported infertility was also significantly more prevalent among women who had higher WWI with an average of 11.21 ± 0.79 (cm/kg(^1/2)).

**Table 1:** Baseline Characteristics of Study Participants (N=3,526).

	Infertility	Control	*p*-value
	N=364	N=3162	
Age, mean ± SD (years)	35.28 ± 7.05	30.95 ± 8.08	<0.001
Ratio of family income to poverty	2.80 ± 1.63	2.61 ± 1.65	0.033
Race (%)			0.080
Mexican American	10.65	12.19	
Other Hispanic	6.09	8.14	
Non-Hispanic White	62.46	55.13	
Non-Hispanic Black	12.18	13.55	
Other Race - Including Multi-Racial	8.61	10.98	
Education lever (%)			0.774
Less than high school	10.73	11.59	
High school	20.03	19.11	
More than high school	69.24	69.30	
Marital status (%)			<0.001
Married or living with partner	77.26	57.46	
Living alone	22.74	42.54	
Smoked at least 100 cigarettes (%)			<0.001
Yes	36.75	29.76	
No	61.25	70.24	
Had at least 12 alcohol drinks/1 year (%)			0.361
Yes	79.17	77.14	
No	20.83	22.86	
Age when first menstrual period occurred (%)			0.012
Age<10	7.69	4.24	
10≤Age<15	79.40	82.70	
15≤Age ≤ 20	12.91	13.06	
Had regular periods in past 12 months			0.007
Yes	84.77	89.31	
No	15.23	10.69	
Ever treated for a pelvic infection/PID (%)			<0.001
Yes	9.14	3.98	
No	90.86	96.02	
Ever taken birth control pills (%)			0.014
Yes	78.63	70.88	
No	21.37	29.22	
Body Mass Index (kg/m^2^)	31.91 ± 9.08	28.80 ± 7.96	<0.001
Waist circumference (cm)	103.22 ± 20.25	94.28 ± 18.07	<0.001
Weight (kg)	85.71 ± 26.06	76.20 ± 22.10	<0.001
A Body Shape Index	80.98 ± 4.65	79.32 ± 4.48	<0.001
Weight-adjusted-waist index	11.21 ± 0.79	10.85 ± 0.81	<0.001

### 3.2 The association between weight-adjusted-waist index and infertility

The association between WWI and infertility is seen in **Table 2**. Our findings indicated that a higher WWI was linked to a higher risk of infertility. Both the crude model and the minimally/fully adjusted model showed evidence of a positive correlation between WWI and infertility. When full adjustments were made, participants with a unit higher WWI had a 42% increased risk of infertility (Model 3: OR = 1.42, 95% CI: 1.22-1.65). After WWI was categorized as tertiles, this association maintained its statistical significance. In comparison to participants in the lowest WWI tertile, individuals in the highest WWI tertile had an importantly 102% increased risk (OR = 2.02, 95% CI: 1.46-2.78; P for trend < 0.001). (**Table 2**).

**Table 2:** Associations between weight-adjusted-waist index and the risk of infertility.

		OR^1^ (95%CI^2^), *P*-value		
		Crude model	Minimally adjusted model	Fully adjusted model
		(Model 1)^3^	(Model 2)^4^	(Model 3)^5^
	N (Infertility)			
Continuous	364	1. 61 (1.42, 1.84) <0.001	1.47 (1.28, 1.69) <0.001	1.42 (1.22, 1.65) <0.001
Categories				
**Tertile 1**	71	**Reference**	**Reference**	**Reference**
Tertile 2	120	1.77 (1.30, 240) <0.001	1.57 (1.15, 2.15) 0.004	1.37 (0.99, 1.91) 0.0588
Tertile 3	173	2.68 (2.01, 3.58) <0.001	2.22 (1.63, 3.00) <0.001	2.02 (1.46, 2.78) 0.01
P for trend		<0.001	<0.001	<0.001

Insensitivity analysis, the weight-adjusted-waist index was converted from a continuous variable to a categorical variable (tertiles).

^1^95% CI: 95% confidence interval.

^2^OR: Odds ratio.

^3^Model 1: Covariates were not adjusted at all.

^4^Model 2: Adjusted for age, and race.

^5^Model 3: Adjusted for age, ratio of family income to poverty, race, education level, marital status, smoked at least 100 cigarettes, had at least 12 alcohol drinks/1 year, age when first menstrual period occurred, ever treated for a pelvic infection/PID, ever taken birth control pills, had regular periods in past 12 months.

**Figure 2:**
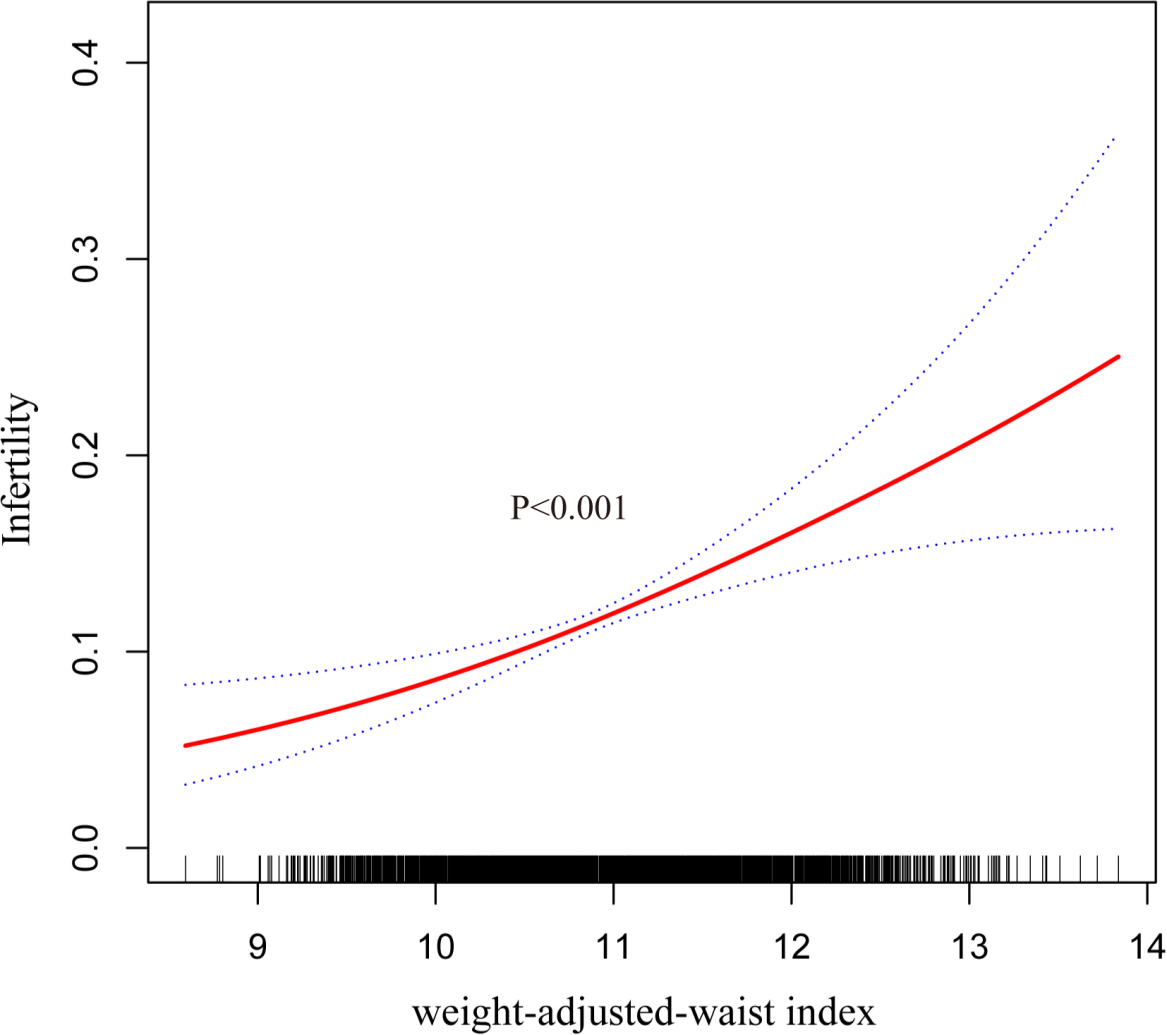
Smooth curve fitting for WWI and infertility.

In addition, we further investigated the relationship between WWI and the risk of infertility using smoothed curve fitting, which showed a positive non-linear relationship (**Figure 2**).

### 3.3 Subgroup analysis

We then conducted subgroup analysis to assess whether the relationship between WWI and infertility was stable across various demographic situations. According to our findings, there was no dependency on the relationship between WWI and infertility. As shown in **Table 3**, none of the stratifications, including race, education lever, marital status, smoke at least 100 cigarettes, had at least 12 alcohol drinks/1 year, age when first menstrual period occurred and had regular periods in past 12 months significantly affected the positive association between WWI and infertility (all P for interaction> 0.05).

**Table 3:** Subgroups analyses of the effect of WWI on infertility.

Subgroups	N		OR (95%CI), *P*-value	*P* for interaction
	Total	Infertility		
**Age**				0.009
Tertile 1	1090	49	1.78 (1.28, 2.49) <0.001	
Tertile 2	1181	122	1.72 (1.36, 2.17), <0.001	
Tertile 3	1255	193	1.16 (0.96, 1.42), 0.129	
**Race**				0.9406
Mexican American	618	61	1.66 (1.14, 2.41), 0.007	
Other Hispanic	369	30	1.45 (0.89, 2.38), 0.136	
Non-Hispanic White	1146	141	1.52 (1.22, 1.89), <0.001	
Non-Hispanic Black	776	75	1.38 (1.05, 1.81), 0.023	
Other Race - Including Multi-Racial	617	57	1.40 (0.95, 2.06), 0.088	
**Education lever**				0.1717
Less than 9th grade	159	13	0.88 (0.43, 1.82), 0.731	
9-11th grade	333	38	2.30 (1.44, 3.66), <0.001	
High school graduate/GED or equivalent	605	71	1.38 (1.01, 1.88), 0.044	
Some college or AA degree	1179	139	1.34 (1.07, 1.67), 0.009	
College graduate or above	863	103	1.49 (1.12, 1.99), 0.006	
**Marital status**				0.5127
Married or living with partner	1369	267	1.36 (1.13, 1.63), 0.001	
Living alone	1770	97	1.49 (1.21, 1.83), <0.001	
**Smoked at least 100 cigarettes**				0.8891
Yes	944	131	1.42 (1.14, 1.78), 0.002	
No	2582	233	1.45 (1.22, 1.72), <0.001	
**Had at least 12 alcohol drinks/1 year**				0.2265
Yes	2467	272	1.55 (1.32, 1.81), <0.001	
No	1059	92	1.27 (0.96, 1.68), 0.088	
**Had regular periods in past 12 months**				0.9038
Yes	3179	326	1.46 (1.27, 1.69), <0.001	
No	347	38	1.42 (0.93, 2.17), 0.100	
**Age when first menstrual period occurred**				0.2797
Age<10	162	28	1.19 (0.74, 1.83), 0.4701	
10≤Age<15	2904	289	1.40 (1.20, 1.64), <0.001	
15≤Age ≤ 20	460	47	1.86 (1.28, 2.69), 0.001	

Subgroup analysis for the association between WWI and infertility. None of the stratifications including race, Education lever, Marital status, smoked at least 100 cigarettes, had at least 12 alcohol drinks/1 year and had regular periods in past 12 months affected the positive association of WWI and infertility.

### 3.4 Weight-adjusted-waist index showed a stronger correlation than other markers of obesity including WC, body mass index and a body shape index for infertility

**Figure 3:**
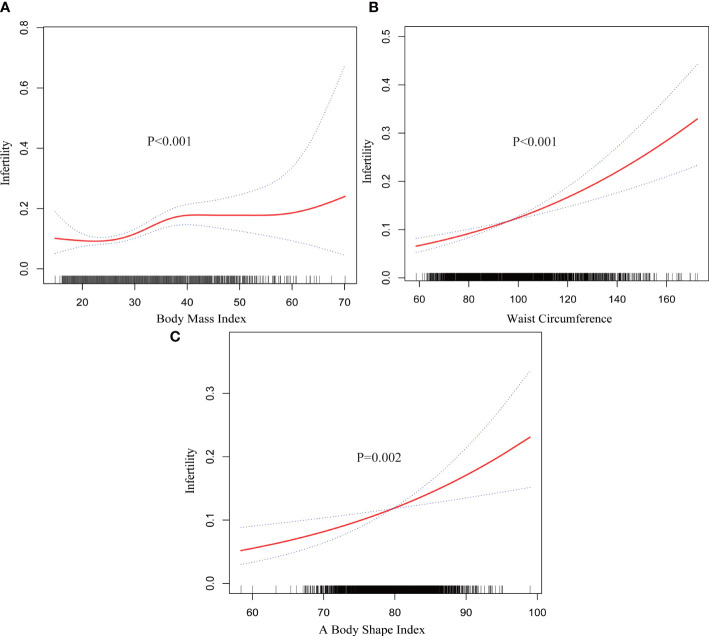
Smooth curve fitting for other markers of obesity and infertility.

Smooth curve fitting revealed non-linearity in the relationships between other markers of obesity including WC, body mass index (BMI) and a body shape index (ABSI) and infertility (**Figure 3**). Then, each interval was fitted using the segmented regression model, and the threshold effect was calculated (**Table 4**).

**Table 4:** Threshold effect analysis of WWI, BMI, WC and ABSI on infertility using a two-piecewise linear regression model.

	WWI	BMI	WC	ABSI
Fitting by standard linear model				
OR^1^ (95%CI^2^) *P*-value	1.42 (1.22, 1.65) <0.001	1.03 (1.02, 1.05) <0.001	1.02 (1.01, 1.02) <0.001	1. 04 (1.02, 1.07) 0.002
Fitting by two-piecewise linear model				
Breakpoint (K)	12.34	19.40	71.60	79.58
OR1 (<K)	1.53 (1.29, 1.82) <0.001	0.66 (0.46, 0.96) 0.03	0.93 (0.78, 1.10) 0.37	1.02 (0.96, 1.08) 0.5417
OR2 (>K)	0.56 (0.19, 1.67) 0.2962	1.04 (1.02, 1.05) <0.001	1.02 (1.01, 1.02) <0. 001	1.06 (1.02, 1.11) 0.0089
OR2/OR1	0.37 (0.11, 1.16) 0.088	1.56 (1.07, 2.27) 0.019	1.10 (0.93, 1.31) 0.288	1.04 (0.96, 1.14) 0.3204
Logarithmic likelihood ratio test *P*-value	0.073	0.034	0.313	0.324

Adjusted for age, ratio of family income to poverty, race, education level, marital status, smoked at least 100 cigarettes, had at least 12 alcohol drinks/1 year, age when first menstrual period occurred, ever treated for a pelvic infection/PID, ever taken birth control pills, had regular periods in past 12 months.

Furthermore, to be able to comment on the OR for WWI in comparison to the OR for other markers of obesity, we calculated their z-scores and used these in the linear models (**Table 5**). Compared with other markers of obesity including WC, body mass index (BMI) and a body shape index (ABSI), WWI had a stronger connection with the risk of infertility (WWI: OR = 1.33; BMI: OR =1.30; WC: OR =1.28; ABSI: OR =1.21), suggesting that it may be a better predictor of the likelihood of infertility than other markers of obesity.

**Table 5:** Associations between the z-scores of markers of obesity and the risk of infertility.

	OR^1^ (95%CI^2^), *P*-value			
z-scores	WWI	BMI	WC	ABSI
Continuous	1.33 (1.18, 1.51) <0.001	1.30 (1.15, 1.45) <0.001	1.28 (1.22, 1.55) <0.001	1.21 (1.07, 1.37) 0.0022

Adjusted for age, ratio of family income to poverty, race, education level, marital status, smoked at least 100 cigarettes, had at least 12 alcohol drinks/1 year, age when first menstrual period occurred, ever treated for a pelvic infection/PID, ever taken birth control pills, had regular periods in past 12 months.

## 4 Discussion

This investigation examined the association between Weight-adjusted-waist index and infertility in non-institutionalized Americans. We discovered that individuals with higher WWI demonstrated an increased probability of infertility in our cross-sectional analysis, which included 3,526 females. Subgroup analysis and interaction test showed that this association between WWI and infertility was stable across various demographic situations. Additionally, we further found a stronger association between WWI and infertility than other markers of obesity, indicating that WWI may be a more accurate predictor of infertility than other obesity-related indicators. According to our research, WWI may be able to predict the prevalence of infertility, and managing obesity as determined by WWI may help to reduce the risk of infertility.

A few studies have investigated the association between central obesity and infertility, and the findings vary from study to study (21–23). According to a prospective cohort of black women (21), greater WC (>84 cm) or WHR (>0.85) was linked to lower fecundity independently of BMI. After adjusting for BMI, a different American prospective cohort discovered that the negative correlations between WC and WHR and fecundity were attenuated (22). In contrast, a multiethnic prospective cohort revealed no correlations for WC, WHR, WHtR or ABSI with fecundability (23). In this population-based study, we found positive associations for WWI, WC and ABSI with infertility. Therefore, it is essential to explore whether abdominal adipocytes have dissimilar hormonal activity in different populations.

To our knowledge, this is the first study to evaluate the association between WWI and infertility, highlighting the relationship between a higher WWI level and increased infertile risks. Compared to conventional equations based on BMI, WWI is a newly developed metric of obesity that more correctly predicts whole-body fat percentage and that has been explored in various fields (15, 24). According to Park et al., WWI was strongly correlated with cardiometabolic morbidity and cardiovascular death among 465,629 participants, which were not evident in BMI (15). Similarly, Ding et al. discovered a non-linear positive correlation between WWI levels and the risk of cardiovascular and all-cause mortality that was independent of BMI and WC (25). Moreover, Xie F et al. uncovered that WWI was strongly linked with higher abdominal aortic calcification (AAC) scores [= 0.95, 95% confidence interval (CI): 0.65-1.25, P< 0.001] and an increased likelihood of severe AAC [odds ratio (OR) = 1.82; 95% CI: 1.20-2.75; P = 0.005] (26). Additionally, Qin Z et al. observed that the prevalence of albuminuria was significantly correlated with WWI (OR = 1.28; BMI: OR = 1.02; WC: OR = 1.01), suggesting that WWI may be a more accurate predictor of the incidence of albuminuria than other obesity indicators like BMI (27). In our analysis, we found out a positive non-linear association of WWI with infertility both in crude model and adjusted model. A dose-response association between WWI and infertility was also revealed by the sensitivity analysis using WWI as a tertile. And more importantly, WWI exhibited a substantially stronger association with the likelihood of infertility than other markers of obesity (WWI: OR = 1.33; BMI: OR =1.30; WC: OR =1.28; ABSI: OR =1.21). In conclusion, it has been widely claimed that WWI might be used as a predictor of obesity-related disorders, and our research supports this claim.

Infertility is a common issue that has an enormous effect on women, families, and communities (28). It is significant to note that obesity may affect the chances of conceiving and delivering a baby. Obesity has a detrimental influence on a person’s ability to conceive, which is mostly attributed to a functional change in the hypothalamic-pituitary-ovarian (HPO) axis (29–31). Other studies have shown that obese women experience longer pregnancy wait times (32, 33). Contrary to popular belief, obese women tend to be infertile even in the absence of ovulatory disorders (34, 35). Obesity also appears to influence the results of assisted reproductive technology (ART), since obese women have smaller oocytes that are less likely to fertilize normally (26, 36, 37). Therefore, it is of great importance to accurately assess obesity to reduce the risk of infertility. However, BMI, the traditional obesity-related anthropometric index, have intrinsic limitations as its inability to discriminate between muscle mass and fat mass (13, 14). WWI, a new obesity index based on weight and WC, may be a complete and superior indication of obesity and might be used to predict problems associated with obesity.

Our study has some limitations to be declared. First, temporality cannot be determined since this research is cross-sectional. Furthermore, despite adjusting for a number of pertinent confounders, we were incapable to completely exclude the influence of other confounding variables, thus our results should be taken with care. Additionally, because our results were based on only one country, it is yet unknown if they apply to nations. Despite these limitations, our research work has a number of advantages. Since sample weights were taken into consideration and national data were used for our study, the results are generally relevant to the whole population of the United States. We used subgroup analyses to confirm the robustness of the regression analysis after adjusting for confounders due to the large sample size. Finally, we further explored that weight-adjusted-waist index showed a stronger correlation than body mass index for infertility.

## 5 Conclusion

This investigation revealed that elevated WWI levels were linked to an increased risk of infertility, and that the association between WWI and infertility was stronger than that between other markers of obesity and infertility, suggesting that WWI may serve as a simple anthropometric index to predict infertility.

## Data availability statement

The original contributions presented in the study are included in the article/supplementary material. Further inquiries can be directed to the corresponding author.

## Ethics statement

The studies involving human participants were reviewed and approved by the National Health and Nutrition Examination Survey. The patients/participants provided their written informed consent to participate in this study.

## Author contributions

ZW and XL designed the research. ZW collected, analyzed the data, and drafted the manuscript. ZW and XL revised the manuscript. All authors contributed to the article and approved the submitted version.
